# Gut microbiota and pediatric eye diseases: current insights, mechanistic underpinnings, and future outlook

**DOI:** 10.3389/fnut.2026.1810321

**Published:** 2026-04-28

**Authors:** Gang Chen, Yulin Zhang, Yixia Zhang, Yun Zhou

**Affiliations:** 1 Department of Pediatric Ophthalmology, Dali Bai Autonomous Prefecture Maternal and Child Health Hospital, Dali, China; 2 Department of Assets and Laboratory Management, Dali University, Dali, China

**Keywords:** gut microbiota, gut-eye axis, immune inflammation, microbiota-based interventions, pediatric ophthalmic diseases, visual development

## Abstract

**Background:**

As a core component of the human microecosystem, the gut microbiota modulates systemic immune-inflammatory responses, metabolic homeostasis, and neural signal transmission via the gut-eye axis. Dysbiosis of the gut microbiota has been closely linked to the onset and progression of multiple ophthalmic disorders in adults. Childhood represents a critical window for both gut microbial colonization and maturation, as well as a formative period for ocular development and visual function establishment.

**Objective:**

This review systematically examines the evidence connecting gut microbiota to several common pediatric ophthalmic diseases, including allergic conjunctivitis, myopia, retinopathy of prematurity, and postoperative inflammation following congenital cataract surgery.

**Methods:**

We conducted a narrative literature review analyzing the potential mechanisms underlying the gut-eye axis, including immune regulation, microbial metabolite signaling, crosstalk between the intestinal barrier and blood-ocular barrier, and neural pathways. A comprehensive search of PubMed, Web of Science, and Scopus databases was performed for literature published from January 2000 to October 2025 using search terms related to gut microbiota and pediatric ophthalmic diseases. Studies were selected based on clinical relevance and contribution to understanding gut-eye axis interactions in pediatric populations.

**Results:**

The review identifies emerging associations between gut microbiota dysbiosis and various pediatric ophthalmic conditions, with evidence supporting immune dysregulation, metabolite imbalance, and barrier dysfunction as potential mechanistic pathways.

**Conclusion:**

Early disruptions to the gut microbiota may exert potential long-term effects on ocular health. This review aims to provide new insights into the etiology, early intervention, and precision management of pediatric ophthalmic diseases.

## 1 Introduction

Visual development in children is highly plastic (1). Early-onset ocular diseases not only compromise immediate visual acuity but may also lead to permanent visual impairment. In recent years, the global prevalence of pediatric ophthalmic conditions has risen continuously. Allergic conjunctivitis affects 10–20% of children (2), while myopia rates among Chinese children and adolescents now exceed 60% (3). Retinopathy of prematurity (ROP) remains one of the leading causes of blindness in preterm infants (4). Traditional research has focused largely on genetic factors, environmental stimuli such as excessive blue light exposure and poor eye care habits, and local pathological changes (5). Comparatively little attention has been paid to the regulatory role of systemic microecological imbalance (6).

The gut microbiota colonizes the gastrointestinal tract and supports nutrient metabolism, immune cell maturation, and pathogen defense (7). Its composition matures and stabilizes primarily within the first three years of life, shaped by delivery mode, feeding practices, antibiotic exposure, and other early-life factors (8). The concept of the gut-organ axis—including the well-established gut-brain axis and gut-lung axis—has revealed far-reaching cross-system regulatory networks between gut microbiota and distant organ functions (9). Building upon these established gut-organ axes, the gut-eye axis has recently emerged as a novel area of investigation. Although research on the gut-eye axis is still in its early stages, it has been implicated in adult ocular diseases such as age-related macular degeneration and dry eye, largely through immune-inflammatory pathways (10). The unique vulnerability of the developing gut microbiota in early life, together with the rapid maturation of the visual system, raises the possibility of a connection between microbial balance and pediatric ocular health (11). However, this relationship remains speculative, and the underlying mechanisms and clinical implications remain poorly defined and incompletely investigated.

This review integrates available evidence to clarify the relationship between gut microbiota and major pediatric ophthalmic diseases. We analyze the mechanistic basis of the gut-eye axis in children, identify gaps in current research, and propose future directions. Our goal is to support the development of microbiota-based interventions and improve the prevention and clinical management of pediatric ocular disorders.

## 2 Colonization patterns and regulatory factors of childhood gut microbiota

### 2.1 Colonization and maturation

The neonatal gut is initially colonized by anaerobic microbes, with delivery mode strongly influencing early community structure (12). Vaginally delivered infants acquire microbes resembling the maternal vaginal tract, dominated by *Lactobacillus* and *Bifidobacterium* species (13). Infants born by cesarean section more frequently harbor environmental strains such as *Staphylococcus* and *Streptococcus* (12). Breastfeeding supports the growth of beneficial bacteria by providing oligosaccharides and other prebiotic components (13). Following weaning, dietary diversification increases microbial diversity, and the community structure gradually approaches an adult-like profile by approximately three years of age (8). disruptions during this period may leave a long-term “microbial imprint” that has been hypothesized to potentially affect the development of multiple organ systems, including the eye, though direct evidence linking such imprinting to ophthalmic outcomes remains limited and requires further investigation (14).

### 2.2 Factors contributing to dysbiosis

Key factors that disrupt childhood gut microbiota include: (i) inappropriate or excessive antibiotic use, which erodes beneficial taxa and enables the expansion of resistant organisms (15); (ii) suboptimal feeding practices, including formula feeding without adequate prebiotic support and high intake of processed foods (13); (iii) over-sanitized environments that reduce natural microbial exposure (14); and (iv) host genetic background that affects microbial colonization and metabolic activity (14). These disturbances have been associated with alterations in microbial composition and function, which may potentially influence ocular health through the gut-eye axis; however, current evidence supporting this connection is primarily associative, and causal relationships remain to be established.

## 3 Gut microbiota in common pediatric ophthalmic diseases

### 3.1 Allergic conjunctivitis

Allergic conjunctivitis is the most frequent ocular surface allergic disease in children, driven largely by exaggerated T-helper 2 (Th2)-type immune responses (16). Gut microbiota helps maintain immune tolerance by promoting regulatory T cell (Treg) differentiation and restraining Th2 polarization (16–18). Microbial imbalance has been associated with disruption of tolerance and increased systemic allergic inflammation in preclinical and observational studies (16). Children with allergic conjunctivitis often show reduced levels of *Bifidobacterium* and *Lactobacillus*, accompanied by increased Enterobacteriaceae and Proteobacteria in small clinical cohort studies (2). Preliminary evidence from limited clinical trials suggests that probiotic supplementation may be associated with lower serum immunoglobulin E (IgE) and reduced symptoms such as ocular itching and conjunctival redness; however, these findings are based on small sample sizes and require further validation (2, 19, 20). These observations are consistent with the hypothesis that gut dysbiosis is associated with allergic conjunctivitis through immune dysregulation, and that probiotics may potentially serve as an adjunctive therapy, although causal relationships have not been established.

### 3.2 Myopia

Myopia arises from a complex interaction of genetic and environmental factors (3, 21, 22). Emerging evidence indicates that gut microbiota may be associated with myopia development through metabolic and immune pathways based on animal model and limited observational studies (23, 24). High-sugar and high-fat diets in childhood alter gut microbial structure, particularly reducing short-chain fatty acid (SCFA)-producing species in preclinical and observational studies (23, 24). The resulting changes in systemic metabolism have been correlated with excessive axial elongation in animal models (23, 24). Studies in germ-free mice (animal models) demonstrate longer ocular axes compared with conventionally colonized animals, and SCFA supplementation can attenuate axial growth in these experimental settings (24, 25). It is important to note that these findings derive from animal models, and their direct applicability to human pediatric populations remains uncertain due to species-specific differences in ocular development, gut microbiota composition, and environmental factors. Clinically, children with myopia display lower gut microbial diversity and reduced expression of genes involved in SCFA synthesis in observational studies (23, 24).

Recent work in animal models has identified that gut-derived indole-3-acetic acid (3-IAA), a tryptophan metabolite, is associated with inhibition of high myopia progression by enhancing type I collagen synthesis in experimental settings (23, 26). *Akkermansia*, which is more abundant in non-myopic children in observational studies, is reduced in those with high myopia and shows inverse correlation with axial length, potentially through supporting 3-IAA production; however, causation has not been established (23). Fecal microbiota transplantation has been shown to mitigate experimental myopia in mice (animal model) via the gut-brain-eye axis (24). These findings, while intriguing, are limited to preclinical animal studies, and extrapolation to pediatric clinical populations requires caution due to differences in species physiology, developmental stages, and environmental exposures. Together, these findings from animal models and limited observational studies suggest microbial metabolites may potentially function as important regulators of ocular growth, though clinical evidence in children remains limited.

### 3.3 Retinopathy of prematurity

ROP is a vasoproliferative disorder of the immature retina, driven by hypoxia-induced vascular endothelial growth factor (VEGF) upregulation and inflammatory activation (4, 27). Preterm infants frequently experience severe gut dysbiosis due to delayed colonization and frequent antibiotic exposure (4, 27). Dysbiosis has been associated with amplified systemic inflammation, including increased levels of tumor necrosis factor-alpha (TNF-*α*), interleukin-6 (IL-6), and other cytokines that have been correlated with retinal vascular pathology in preclinical and observational studies (4, 27–29). Infants with ROP often exhibit reduced Bacteroidetes and elevated Clostridia in small clinical cohort studies (4, 27). Inflammatory markers show correlation with microbial structure in these observational studies, though causation remains unproven (4). Limited studies suggest that probiotic interventions may be associated with reduced systemic inflammation in preterm infants and could potentially lower ROP risk; however, clinical evidence remains limited by small sample sizes, variability in probiotic strains and dosing, inconsistent study designs, and lack of large randomized controlled trials (4).

### 3.4 Postoperative inflammation in congenital cataracts

Congenital cataract is a major cause of childhood blindness. Postoperative complications, including intraocular inflammation and posterior capsule opacification, are closely linked to excessive immune activation (10). Gut microbiota has been proposed to modulate postoperative inflammatory tone by shaping systemic immune tolerance based on mechanistic hypotheses and limited observational data (10). Children with more favorable microbial profiles have been observed to have higher anti-inflammatory cytokine levels (e.g., interleukin-10 (IL-10)) and shorter periods of intraocular inflammation in small clinical cohorts (10). These associations do not establish causation. By contrast, dysbiosis has been associated with higher complication rates in observational studies (10). Gut microbial status has been proposed as a potential prognostic indicator for congenital cataract surgery, and targeted microbiota-based support might potentially improve postoperative recovery; however, these suggestions are based on preliminary observational data and mechanistic hypotheses rather than established clinical evidence from randomized controlled trials.

## 4 Mechanisms of the gut-eye axis in pediatric ocular diseases

### 4.1 Immune regulation

The gut represents the body’s largest immune interface (17). Gut microbiota interacts with the mucosal immune system to regulate the balance of Treg, T-helper 1 (Th1), and Th2 cells, maintaining systemic immune homeostasis (16–18, 30). This immune-regulatory function of gut microbiota is well established in both animal models and human studies, though direct evidence specifically in pediatric ocular disease remains limited. Dysbiosis reduces beneficial taxa, impairs immune tolerance, and triggers the release of pro-inflammatory cytokines including TNF-α, IL-6, and interleukin-17 (IL-17) (28, 29, 31). These molecules enter the circulation and may affect ocular tissues, potentially promoting inflammation in conditions such as allergic conjunctivitis and ROP (2, 4, 16). The direct causal relationship between circulating cytokines and pediatric ocular inflammation represents a proposed mechanism that requires further experimental validation. In some cases, microbial-induced antibodies have been hypothesized to cross-react with ocular antigens and potentially exacerbate tissue damage (10). This molecular mimicry hypothesis remains speculative and is primarily supported by indirect evidence from adult autoimmune conditions rather than direct pediatric ocular studies.

### 4.2 Metabolite-mediated signaling

Gut microbiota produces a wide range of metabolites, including SCFAs, bile acids, and lipopolysaccharides (LPS), which can reach the eye through the bloodstream (10, 32, 33). SCFAs exert anti-inflammatory effects, suppress intraocular inflammation, and have been shown to help regulate retinal vascular endothelial proliferation in experimental models, with proposed roles in both myopia and ROP (17, 18, 23, 34–37). The evidence supporting SCFA-mediated ocular effects is moderately established in animal studies, though translation to pediatric populations requires further investigation. LPS promotes inflammation and compromises blood-ocular barrier integrity in experimental settings (38, 39). Bile acids act through nuclear receptor pathways to potentially influence ocular metabolism and development (32, 33, 40–49). The role of bile acids in pediatric ocular physiology remains largely hypothetical, with most supporting evidence derived from adult metabolic studies or *in vitro* experiments. Notably, 3-IAA promotes COL1A1 transcription by enhancing specificity protein 1 (SP1) recruitment to the promoter region, thereby inhibiting axial elongation in high myopia (23, 26). This specific pathway has been demonstrated in experimental myopia models and represents one of the more mechanistically well-characterized examples of microbial metabolite action in ocular disease, though clinical validation in pediatric populations is still needed. This pathway underscores the importance of microbial metabolites in the gut-eye axis.

### 4.3 Barrier crosstalk

The intestinal barrier and blood-ocular barrier function in a coordinated manner represents a proposed conceptual framework (50–53). While barrier dysfunction is well documented in various disease states, the direct mechanistic linkage between intestinal and ocular barrier function remains incompletely understood and is primarily supported by circumstantial evidence. Gut dysbiosis impairs intestinal mucosal integrity, allowing food antigens, pathogens, and microbial products to enter the circulation and trigger low-grade systemic inflammation (38, 39, 50). This inflammatory state may disrupt the blood-ocular barrier, rendering ocular tissues more susceptible to inflammation and damage (10). The hypothesis that systemic inflammation secondary to gut barrier dysfunction directly compromises the blood-ocular barrier is plausible but remains to be definitively demonstrated in pediatric ocular disease models. In turn, ocular inflammation has been hypothesized to feed back to alter gut microbial composition via neuroendocrine pathways, creating a bidirectional gut-eye regulatory loop (10). This proposed bidirectional communication represents a speculative extension of the gut-eye axis concept, with limited direct experimental evidence currently available.

### 4.4 Neural pathways

Gut microbiota has been proposed to signal to the eye through the gut-brain-eye axis (9, 54–56). This neural pathway mechanism remains largely hypothetical in the context of ocular disease and is primarily inferred from studies of gut-brain communication in other physiological systems. Microbial metabolites may stimulate intestinal neural endings, which transmit signals to the central nervous system via the vagus nerve (9, 54). These signals have been hypothesized to modulate ocular neural function, vascular tone, axial growth, and retinal perfusion (10). The specific effects of vagally-mediated signals on ocular physiology remain speculative and are not yet supported by direct experimental evidence. Microbiota-driven central inflammation may potentially influence visual pathway development (57–59). This proposed mechanism, while biologically plausible, lacks direct empirical validation in pediatric ophthalmic conditions. The full scope of this regulation in pediatric myopia and congenital optic nerve disorders warrants further investigation.

### 4.5 Schematic overview of gut-eye axis mechanisms

**Figure 1:**
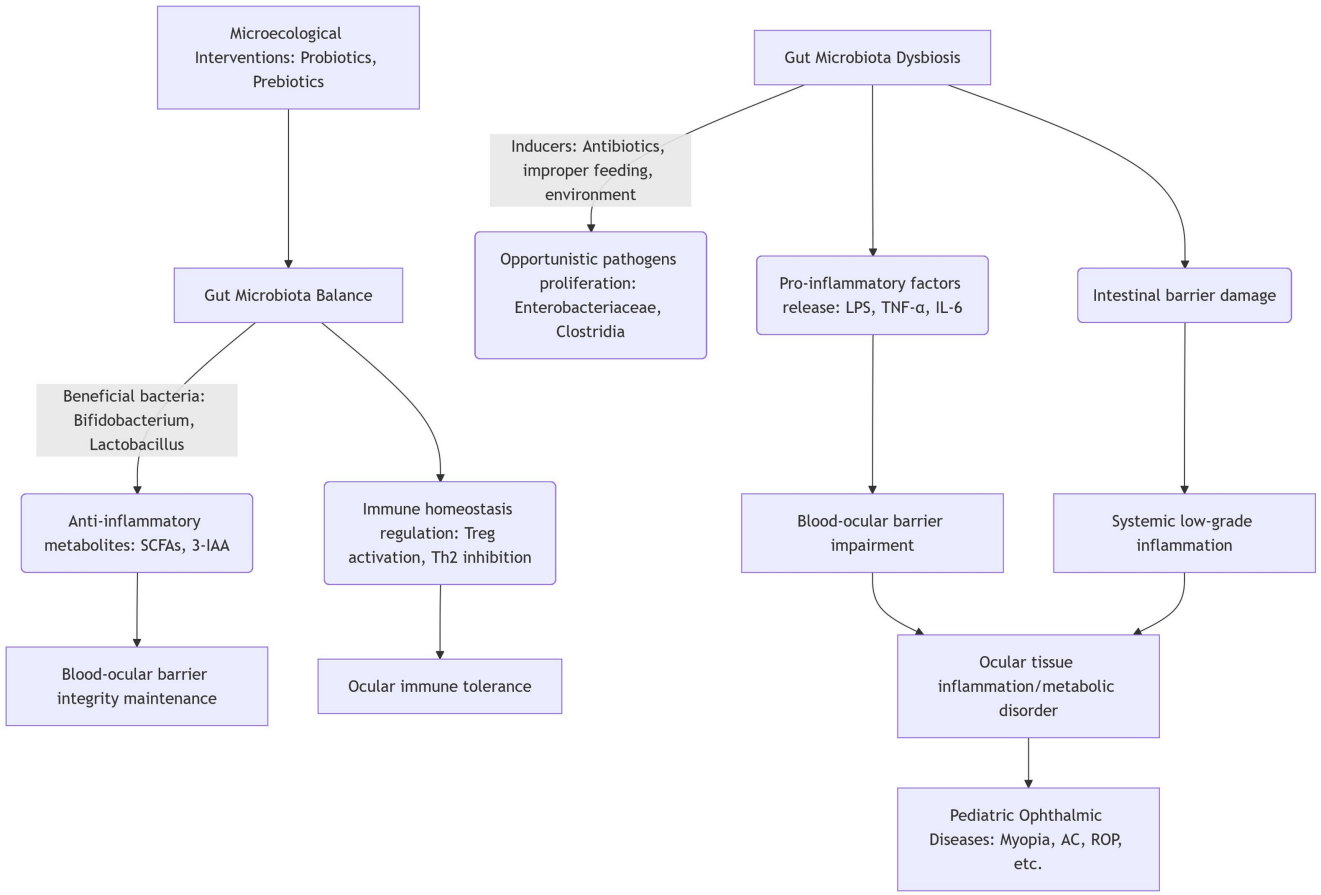
Comprehensive mechanistic overview of the gut-eye axis in pediatric ophthalmic diseases, illustrating the transition from physiological homeostasis to pathological dysregulation through four interconnected pathways. Physiological state (left panel–homeostasis): Beneficial bacteria (e.g., *Bifidobacterium*, *Lactobacillus*) maintain eubiosis through: (1) Metabolic production: Synthesis of SCFAs (acetate, propionate, butyrate) via fermentation of dietary fibers. Butyrate serves as the primary energy source for colonocytes and enhances tight junction protein expression (occludin, claudin-1, ZO-1). Indole-3-acetic acid (3-IAA) derived from tryptophan metabolism activates the aryl hydrocarbon receptor (AhR), promoting IL-22 production and barrier maintenance. (2) Immune modulation: Induction of regulatory T cells (Tregs) through dendritic cell-mediated antigen presentation and SCFA-mediated HDAC inhibition. Tregs suppress effector T cells (Th1, Th2, Th17) via IL-10 and TGF-β secretion, maintaining systemic immune tolerance. (3) Barrier integrity: Enhanced mucus production (MUC2), antimicrobial peptide secretion (RegIIIγ, β-defensins), and intact tight junctions prevent pathogen translocation. These mechanisms collectively preserve blood-ocular barrier function through reduced systemic inflammation and maintained endothelial integrity. Pathological state (right panel–dysbiosis): Disruption of microbial balance triggered by antibiotics, high-fat/high-sugar diets, or environmental stressors leads to: (1) Pathogen expansion: Overgrowth of opportunistic bacteria (e.g., *Enterobacteriaceae*, *Clostridia*) and reduction of beneficial species, altering metabolic output (↓SCFAs, ↑toxic metabolites). (2) Barrier disruption: Reduced tight junction expression increases intestinal permeability (“leaky gut”), enabling translocation of lipopolysaccharides (LPS), peptidoglycans, and unmetabolized dietary components into the bloodstream. (3) Systemic inflammation: LPS binding to TLR4 on immune cells activates NF-κB and MAPK pathways, triggering release of TNF-α, IL-6, IL-1β, and IL-17. These cytokines: (a) Further compromise intestinal barrier via MMP activation; (b) disrupt blood-ocular barrier integrity by downregulating endothelial tight junctions; (c) promote ocular tissue inflammation and metabolic dysfunction. (4) Disease manifestation: The inflammatory and metabolic cascade contributes to specific pediatric ocular pathologies—myopia (altered scleral remodeling), allergic conjunctivitis (Th2-mediated eosinophilic inflammation), ROP (VEGF-driven pathological angiogenesis), and postoperative inflammation (exaggerated immune response). Therapeutic intervention points: Microbiota-based interventions (probiotics, prebiotics, synbiotics) target multiple stages of this pathway: (1) Direct colonization with beneficial strains to restore eubiosis; (2) enhanced SCFA production to support barrier function and immune regulation; (3) competitive exclusion of pathogens; (4) modulation of host immune responses toward tolerance. The multi-target approach explains the potential adjunctive therapeutic value, though clinical efficacy in pediatric ophthalmology requires further validation.

To summarize the complex regulatory network of the gut-eye axis in pediatric ophthalmic diseases, we have integrated the aforementioned mechanisms (immune regulation, metabolite signaling, barrier crosstalk, and neural pathways) into a conceptual framework. It should be noted that this framework includes both well-established mechanisms (particularly those involving immune regulation and SCFA signaling) and more speculative pathways (including neural signaling and bidirectional barrier communication) for which direct experimental evidence remains limited. This framework distinguishes between the physiological state of gut microbiota balance and the pathological state of dysbiosis, illustrating how gut microbial changes may affect ocular health through multiple pathways and highlighting the potential role of microbiota-based interventions in interrupting pathological processes (Figure 1).

### 4.6 Summary of gut microbiota changes, mechanisms, and interventions

Based on the preceding analysis of the association between gut microbiota and each pediatric ophthalmic disease, we have summarized the core information in Table 1 for concise reference. This table systematically collates the characteristic changes in gut microbiota across different pediatric ophthalmic diseases, the corresponding core regulatory mechanisms, and potential microbiota-based interventions, providing a convenient reference for clinical researchers and practitioners to quickly grasp key advances in this field. Readers should note that the mechanisms presented span a range of evidentiary support, from well-established immune and metabolic pathways to more hypothetical neural and barrier-mediated processes requiring further investigation.

**Table 1:** Summary of gut microbiota changes, mechanisms, potential interventions, and representative references in common pediatric ophthalmic diseases.

Pediatric ocular disease	Gut microbiota changes	Core mechanisms	Potential microbiota-based interventions	References
Allergic conjunctivitis	↓ Bifidobacterium, Lactobacillus; ↑ Enterobacteriaceae, Proteobacteria	Th2 polarization; impaired Treg differentiation; broken immune tolerance	Probiotics (preliminary evidence); prebiotics (investigational)	(2, 16–20)
Myopia	↓ Microbial diversity; ↓ SCFA-producing species; ↓ Akkermansia	Metabolite imbalance (↓ SCFAs, ↓ 3-IAA); altered axial elongation via collagen synthesis	SCFA-related probiotics (investigational); dietary modification (preliminary evidence)	(23–26)
Retinopathy of prematurity	↓ Bacteroidetes; ↑ Clostridia; delayed colonization	Systemic inflammation (↑ TNF-α, IL-6); VEGF overexpression; blood-retinal barrier disruption	Early probiotics in preterm infants (preliminary evidence)	(4, 27–29)
Congenital cataract postoperative inflammation	Reduced beneficial taxa; immune regulatory imbalance; ↓ anti-inflammatory potential	Excessive postoperative inflammation; compromised blood-ocular barrier; ↓ IL-10	Perioperative probiotic support (investigational)	(10)

SCFAs, short-chain fatty acids; 3-IAA, indole-3-acetic acid; VEGF, vascular endothelial growth factor; Th2, T-helper 2 cell; Treg, regulatory T cell; TNF-α, tumor necrosis factor-alpha; IL-6, interleukin-6; IL-10, interleukin-10.

## 5 Limitations and challenges

Research linking gut microbiota to pediatric ophthalmic diseases remains in an early phase. Importantly, the majority of evidence discussed in this review remains associative rather than mechanistically confirmed in pediatric populations, with most findings derived from observational studies that cannot establish causal relationships. Several important limitations exist:

1. Most clinical studies are cross-sectional with small sample sizes and short follow-up periods, making causal inference difficult. These methodological constraints significantly limit the ability to draw definitive conclusions about microbiota-ophthalmic disease relationships in children.
2. The composition of childhood gut microbiota is affected by numerous confounding variables including feeding practices, antibiotic exposure, and environmental factors, which are difficult to fully control in clinical studies (14, 15). This complexity presents substantial challenges for isolating specific microbiota-disease associations.
3. The precise, disease-specific molecular pathways of the pediatric gut-eye axis remain incompletely characterized. Mechanistic studies specifically investigating how gut microbial alterations directly influence ocular pathophysiology in pediatric patients are scarce, and most proposed mechanisms remain theoretical or extrapolated from adult or animal models.
4. Standards for probiotic or prebiotic dosing, timing, and formulation are lacking, and clinical efficacy requires rigorous validation (2, 19, 20).
5. Ethical and practical challenges in obtaining pediatric ocular tissues limit direct study of local responses to microbial signals.

Probiotic Research Limitations: Despite the promising therapeutic potential of probiotics and microecological interventions for pediatric ophthalmic conditions, several critical limitations must be acknowledged. First, substantial variability exists in the probiotic strains employed across studies, with different species, subspecies, and formulations yielding inconsistent biological effects. Second, the absence of standardized dosing protocols—including optimal colony-forming units, administration frequency, and treatment duration—complicates direct comparison of clinical outcomes. Third, significant heterogeneity in study design, including variations in participant populations, outcome measures, and follow-up periods, limits the generalizability of findings. Fourth, the current literature reports inconsistent clinical outcomes, with some studies demonstrating significant benefits while others show minimal or no therapeutic effect. These limitations collectively underscore the urgent need for well-designed, large-scale randomized controlled trials with standardized protocols to establish evidence-based recommendations for probiotic interventions in pediatric ophthalmology.

## 6 Future directions and clinical perspectives

### 6.1 Basic research

Future studies should employ gnotobiotic and disease-specific animal models to establish causal relationships and clarify age-specific mechanisms (24, 25). The timing of critical windows for microbial colonization and ocular development should be mapped to identify opportunities for reversible early interventions (8, 11). Discovery of microbial and metabolite biomarkers will support early risk stratification (23, 24).

### 6.2 Clinical research

Large prospective cohort studies are needed to track longitudinal changes in gut microbiota and the incidence of pediatric ocular diseases, enabling predictive modeling (3, 11). Randomized controlled trials are required to validate the efficacy of probiotics, prebiotics, and fecal microbiota transplantation, and to define optimal protocols (2, 19, 20). The clinical utility of microbial signatures for diagnosis, monitoring, and prognosis should be explored to advance precision pediatric ophthalmology.

### 6.3 Clinical translation

The development of targeted microbiota-based products for the prevention and adjunctive treatment of myopia and allergic conjunctivitis warrants further investigation (2, 23). Exploring the incorporation of gut microbiota assessment into ROP screening may represent a potential avenue for future research toward early intervention (4, 27). Investigating whether perioperative microbial modulation could reduce inflammation and improve outcomes following congenital cataract surgery remains an area for future study (10). Exploring public health strategies promoting balanced nutrition and rational antibiotic use as potential approaches to preserve gut microbial health and support lifelong visual development may be valuable for future research (14, 15).

## 7 Conclusion

**Figure 2:**
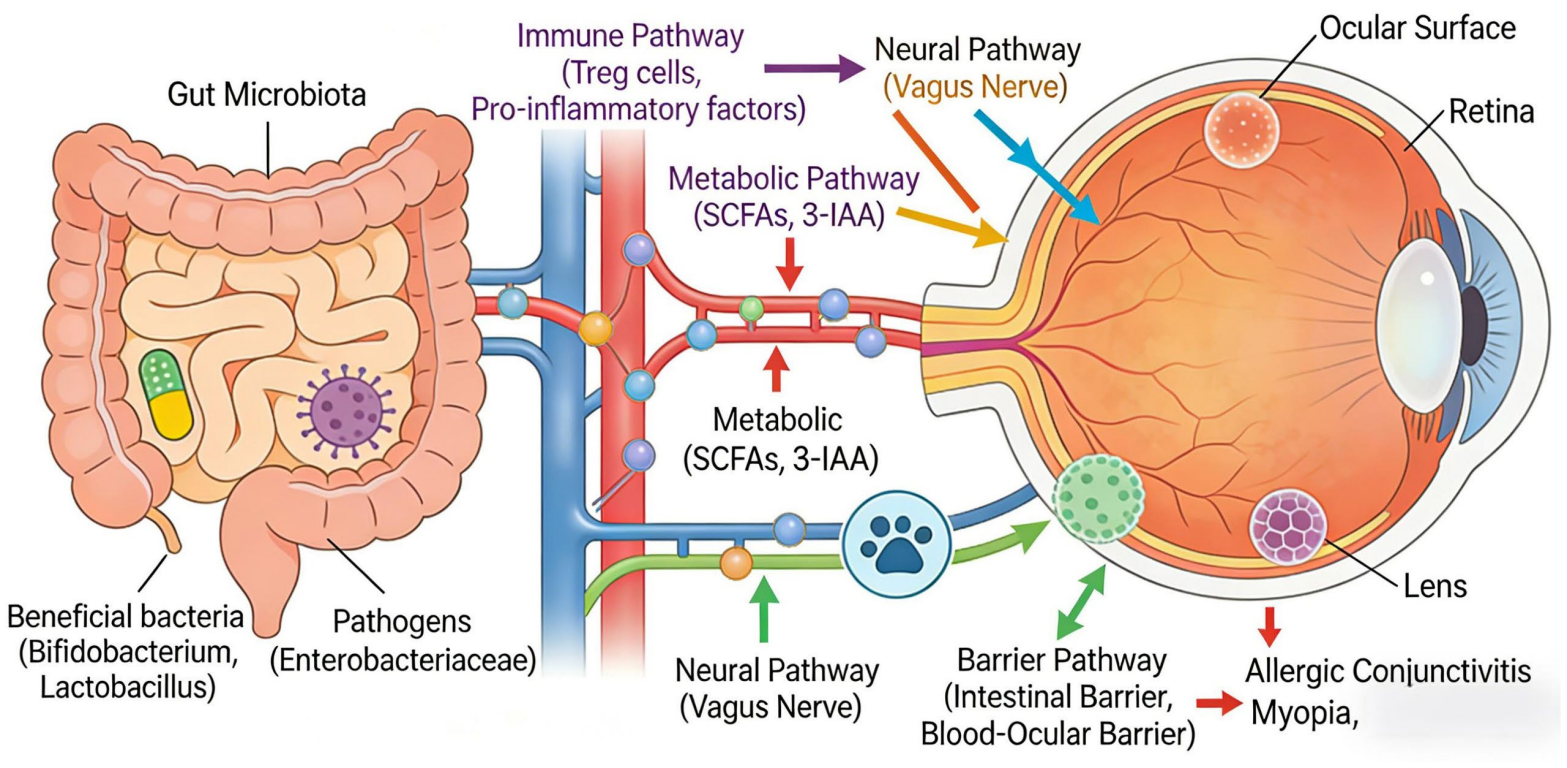
Schematic diagram illustrating the multidimensional Gut-Eye Axis regulatory network in pediatric ophthalmic diseases. This diagram depicts the complex bidirectional communication between gut microbiota and ocular health through four interconnected mechanistic pathways. Pathway interactions and crosstalk: (1) Immune-metabolic crosstalk: SCFAs (particularly butyrate) produced by beneficial bacteria inhibit histone deacetylases (HDACs) in immune cells, enhancing Treg differentiation while suppressing Th1/Th17 responses. Simultaneously, tryptophan metabolites (e.g., 3-IAA) activate the aryl hydrocarbon receptor (AhR) pathway, modulating both immune cell function and collagen synthesis in the sclera. (2) Barrier-immune interplay: Intestinal barrier disruption allows translocation of pathogen-associated molecular patterns (PAMPs) such as LPS, which activate pattern recognition receptors (PRRs) on immune cells, triggering NF-κB and MAPK signaling cascades that amplify systemic inflammation. This inflammatory milieu further compromises both intestinal and blood-ocular barrier integrity through cytokine-mediated downregulation of tight junction proteins (occludin, claudins, ZO-1). (3) Neural-immune communication: The vagus nerve mediates cholinergic anti-inflammatory signaling, where acetylcholine release inhibits NF-κB activation in macrophages and reduces pro-inflammatory cytokine production. Gut microbial metabolites may modulate this neural-immune interface by influencing enteric nervous system activity and vagal afferent signaling. (4) Bidirectional Gut-Eye signaling: Ocular inflammation generates neuroendocrine signals (e.g., cortisol, catecholamines) that alter gut motility, secretion, and permeability, potentially exacerbating dysbiosis and creating self-perpetuating pathological cycles. Disease-specific pathway activation: In myopia, dopamine signaling in the retina interacts with microbial metabolite-mediated collagen synthesis pathways. For allergic conjunctivitis, IL-33 and TSLP (thymic stromal lymphopoietin) from ocular surface epithelium may feedback to influence gut immune responses. In ROP, VEGF-driven angiogenic dysregulation correlates with microbially-modulated systemic inflammation levels. Intervention targets: Microbiota-based interventions (probiotics, prebiotics) target multiple nodes within this regulatory network: (a) Restoration of beneficial bacterial populations enhances SCFA and 3-IAA production; (b) Improved barrier function reduces PAMP translocation; (c) Enhanced Treg differentiation suppresses pathogenic Th2/Th17 responses; (d) Vagal signaling modulation may optimize ocular blood flow and neural function. The multi-target nature of these interventions underscores their potential as adjunctive therapies, though clinical validation in pediatric populations remains ongoing.

Gut microbiota dysbiosis in early childhood has been implicated in the development and progression of multiple pediatric ophthalmic diseases, though this evidence base remains emerging. The gut-eye axis integrates immune regulation, metabolite signaling, barrier crosstalk, and neural communication to link intestinal health with ocular function (Figure 2) (10). While existing studies have established meaningful associations, important gaps remain in mechanistic understanding and clinical translation, and the field remains exploratory. If validated through continued research, advances in microbiome technology could eventually deepen investigation into the gut-eye axis and may potentially open new avenues for the prevention and treatment of pediatric ocular diseases; however, such applications remain investigational at this time. Should these hypotheses be confirmed, such developments could have profound implications for children’s visual health worldwide.
